# Current and emerging therapeutic strategies for Fanconi anemia

**DOI:** 10.1186/1877-6566-6-1

**Published:** 2012-03-09

**Authors:** Pallavi Shukla, Kanjaksha Ghosh, Babu R Vundinti

**Affiliations:** National Institute of Immunohaematology (NIIH), Mumbai, India

**Keywords:** Fanconi Anemia, Androgens, Haematopoitic growth factors, Haematopoietic stem cell transplantation, Gene therapy, Small molecule intervention, DNA repair pathway

## Abstract

Fanconi Anemia (FA) is a rare disorder with incidence of 1in 350,000 births. It is characterized by progressive bone marrow failure leading to death of many patients in their childhood while development of cancer at later stages of life in some. The treatment of FA is still a medical challenge. Current treatments of FA include androgen administration, hematopoietic growth factors administration and hematopoietic stem cell transplantation (HSCT). Clinical gene therapy trials are still ongoing. The partial success of current therapies has renewed interest in the search for new treatments. Generation of patient-specific induced pluripotent stem (iPS) has shown promising results for cell and gene based therapy. Small molecule interventions have been observed to delay tumor onset in FA. Tumors deficient in FA pathway can be treated by profiling of DNA repair pathway through synthetic lethality mechanism. Targeting toll-like receptor 8 (TLR8) dependent TNFα overexpression is yet another upcoming therapeutic approach to treat FA patients. In conclusion, in the present scenario of treatments available for FA, a proper algorithm of treatment decisions must be followed for better management of FA patients and to ensure their increased survival. Innovative therapeutic approaches that can prevent both anemia and cancer should be developed for more effective treatment of FA.

**Electronic supplementary material:**

The online version of this article (doi:10.1186/1877-6566-6-1) contains supplementary material, which is available to authorized users.

## Introduction

**Figure 1:**
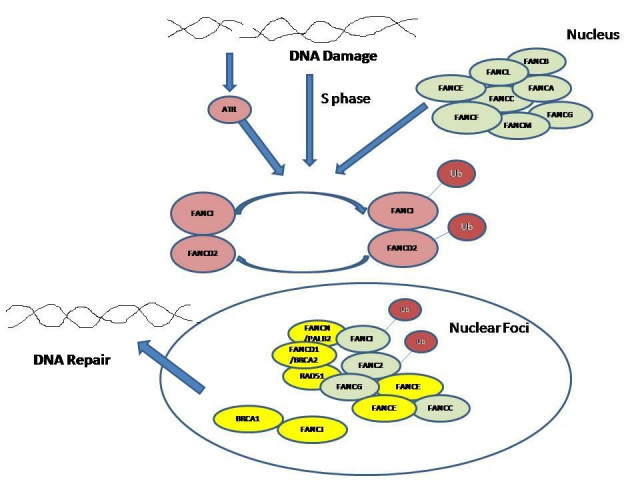
**FA/BRCA pathway showing role of Fanconi Anemia proteins in DNA damage repair**.

Fanconi anemia (FA) is an autosomal recessive disorder (OMIM 227650). It is characterized by bone marrow failure (aplastic anemia), developmental delay, physical abnormalities such as short stature; microcephaly; abnormal skin pigmentation; malformations of skeletal system, limbs, eyes, ears, kidneys and urinary tract, heart, genitalia, gastrointestinal system and central nervous system; and increased incidence of solid tumors and leukemias (Auerbach et al. 2001; D'Andrea and Grompe 1997). To date, around 14 genes are responsible for known complementation FA groups: A, B, C, D1 [BRCA2], D2, E, F, G, I, J [BRIP1], L, M, N [PALB2] and P [SLX4] are known. There are three other genes RAD51C, DDX11, FAAP20 which are involved in the pathway, but not formally designated as FA genes (Svahn and Dufour 2011). FA proteins play an important role in repair of DNA damage. When DNA damage occurs, the FA proteins, A, B, C, E, F, G, I, L, and M at upstream of the pathway monoubiquitinate FANCD2 protein, resulting in targeting of protein in nuclear foci. FANCD2 then interacts with BRCA1 and other DNA damage response proteins downstream of the FA pathway such as BRCA2, RAD51 and NBS to repair the damage (Wang et al. 2004) (Figure 1). Alterations in FA-pathway proteins has also been reported in various types of cancers such as breast cancer, ovarian cancer, lung cancer, cervical cancer, pancreatic cancer etc (Dhillon et al. 2004; Marsit et al. 2004, Narayan et al. 2004; Taniguchi et al. 2003; van Der Heijden et al. 2003). Thus, FA proteins take part in DNA damage repair directly or indirectly. Indirect role of FA proteins in repairing the DNA damage is by triggering a cell cycle checkpoint (Sala-Trepat et al. 2000).

FA proteins are also known to interact with other proteins such as FANCA and FANCC with α spectrin II; FANCC with cytochrome P-450 reductase and with glutathione S-transferase, Hsp70, STAT1; and FANCG with cytochrome P-450 2E1 (McMahon et al. 1999; Pagano and Youssoufian, 2003; Pang et al. 2000; Futaki et al. 2002). Interactions with these proteins suggest the diverse role of FA proteins in a number of cellular processes, such as DNA synthesis, cell cycle progression, gene expression, signal transduction, cell growth and differentiation, and protection against oxidative DNA damage. This could possibly explain some of the diverse cellular defects such as cell cycle defects, aberrant induction of apoptosis, and clinical heterogeneity reported in FA.

Presently the available treatments for FA are androgen administration, administration of hematopoietic growth factors and bone marrow transplantation. Clinical Gene therapy trials still have a long way to go. Small molecule intervention, controlling TNFα overproduction and profiling of DNA damage repair pathway are the new emerging therapies under investigation. This review highlights the current treatments available for FA and discusses new and emerging therapeutic strategies that can be exploited for the treatment of FA in future.

## Current treatments

### Androgen administration

Androgens are male hormones (many aren't artificial) that stimulate production of red blood cell count, platelets, and white blood cells in some FA patients. Oxymetholone, a 17-a-alkylated androgen is the most common androgen used in the treatment of FA patients. Use of androgens has many pitfalls. Androgens are not a permanent cure, but only prolong the life of patients. Patients response to androgens only if the treatment is started at early stage. Though 70% of the patients respond to androgens, the response is slow, drug dependent and incomplete. Other limitations include several side effects such as masculinisation, acne, growth spurt and premature closing of epiphysis and risk to liver tumours (Moldvay et al. 1991; Mulvihill et al. 1975; Ozenne et al. 2008; Touraine et al. 1993). Thus, in case of androgen therapy, follow up of patient has to be done for evaluation of liver function test (enzymes, bilirubin) and ultrasound examination. Androgens are also reported to adversely affect the outcome of subsequent haematopoietic stem cell transplantation (HSCT) in some studies (Dufour et al. 2008). In view of these limitations of androgens, the treatment is recommended for FA patients with aplastic anemia for whom there is no acceptable hematopoietic stem cell transplant donor available (Velazquez and Alter 2004).

### Hematopoietic growth factors

Hematopoietic growth factors (HGFs) bind with high affinity to specific receptors expressed on the surface of the target cells and stimulate primitive hematopoietic stem and progenitor cells as well as activate some mature cells.

Several growth factors such as recombinant (rh) interleukin (IL)-3 and granulocyte-macrophage colony-stimulating factor (rhGM-CSF), granulocyte colony-stimulating factor (rhG-CSF), have been used in human trials with FA patients (Gillio and Gabrilove 1993; Guinan et al. 1994; Rackoff et al. 1996). Administration of rhGM-CSF raises the neutrophil count in most pancytopenic patients, but the platelet count and haemoglobin concentration are generally unaffected. Thus, rhGM-CSF therapy can be used for stabilizing neutropenic complications of the disease (Rackoff et al. 1996). A study on fancc^-/-^ mouse model showed that the short term use of G-CSF either alone or in combination with erythropoietin increased the peripheral blood count and delays the mitomycin C (MMC) induced bone marrow failure, but its long term administration leads to increased sensitivity to MMC and bone marrow hypoplasia. A study demonstrated that FANCC binds to STAT1 and facilitates its activation by gamma interferon and hematopoietic growth factors (Pang et al. 2000).

Due to the limited success of the preliminary results, clinical trials of HGFs administration have not been carried out so far. Moreover, cytokine use is not recommended for patients with a clonal cytogenetic abnormality. Further studies are therefore needed to search novel cytokines and to establish the long-term efficacy of HGFs and their impact on progression of FA to acute myeloid leukaemia (AML) or myelodysplasia.

### Bone marrow transplantation

Bone marrow transplantation (BMT) or HSCT is the only curative therapy for marrow failure in FA patients. Early attempts with BMT for the treatment of marrow failure had a limited success as excessive regimen-related toxicity, graft failure, and severe acute graft-versus-host disease (GVHD) leads to high mortality rate (Davies et al. 1996; Gluckman et al. 1995; Wagner et al. 2003). There is also a risk of developing solid tumours at later stages after the hematopoietic stem cell transplantation. As FA cells are hypersensitive to DNA cross linking agents, FA patients exhibit increased sensitivity to exposure to use of cyclophosphamide (CY) or irradiation. Thus different chemotherapy and radiation regimens were proposed to lower the adverse effect in the BMT treatment on FA patients. Gluckman et al in 1983 demonstrated that low-dose cyclophosphamide and thoracoabdominal irradiation be used as the conditioning regimen for FA patients for BMT treatment (Gluckman et al. 1983). Use of low-dose cyclophosphamide and thoracoabdominal irradiation was also supported by other study in which 58% survival of patients over 100 months was reported (Socié et al. 1998). However this reduced intensity conditioning regimen was found to be unsuccessful for consistent engraftment in recipients of unrelated donor BMT (Gluckman et al. 1983, Guardiola et al. 2000; Wagner et al. 2003). Later MacMillan et al showed that the high rate of graft failure occurs in patients with T cell mosaicism and a standard therapy regimen consisting of cyclophosphamide (CY), total body irradiation (TBI), and anti-thymocyte globulin (ATG) was insufficient to eradicate DEB-resistant T cells (MacMillan et al. 2011). Further studies confirmed that the use of fludarabine in the preparative therapy increases the overall survival rate of patients undergoing BMT (Kapelushnik et al. 1997; MacMillan et al. 2005; Wagner et al. 2007). Addition of fludarabine in the therapy regimen was also reported to overcome the high frequency of graft rejection due to incomplete ablation of DEB-resistant lymphocytes in somatic mosaic FA patients (Wagner et al. 2007).

It was also demonstrated that in case of unrelated donor BMT, in which GVHD is the major limitation, the combined therapy of fludarabine-based preparatory regimens and T-cell depletion reduces the risk of GVHD and increases the overall survival (Bitan et al. 2006; Drobyski 2000). Because GVHD has been associated with an increased risk of malignancy in patients, strategies to reduce the risk of GVHD and improve overall survival have been developed. In an attempt to decrease the risk and severity of regimen-related toxicities, Pasquini and group in 2008 studied outcomes of HSCT in 148 FA patients after irradiation and non-irradiation regimens and found that hematopoietic recovery, acute and chronic GVHD and mortality were similar in both regimens (Pasquini et al. 2008). Gluckman and Wagner in the recent review have proposed that HLA-identical sibling hematopoietic stem cell transplantation should be performed as first-line therapy, without first using androgens or corticosteroids due to their side effects. They also proposed that the best conditioning regimen is combination of fludarabine-containing regimens with low-dose cyclophosphamide (CY) or Bu and antithymocyte globulin (ATG). This regimen was found to overcome rejection in patients receiving multiple transfusions, limit early and late toxicities and minimize risk of GVHD (Gluckman and Wagner 2008). Recently, MacMillan et al demonstrated that sufficient engraftment is possible with minimum of 300 cGy Total body Irradiation (TBI) in recipients of cyclophosphamide- fludarabine-antithymocyte globulin therapy and HLA matched or mismatched T cell-depleted alternative donor BM or unmanipulated umbilical cord blood (MacMillan et al. 2009).

Though the HSCT has been successful in increasing the overall survival rate of patients but unfortunately occurrence of cancer has been observed in long-term survivors. In a study of a retrospective cohort of 145 North American FA patients, the crude rate for solid tumors prior to BMT, AML, or death was 0.7% per year, whereas the rate was 1.99% after BMT (Rosenberg et al. 2003). In 2005, Rosenberg reported that the incidence of squamous cell carcinoma (SCC) was 4.4-fold higher in FA patients who received transplants than in those who did not, and SCCs occurred at significantly younger ages in the former (Rosenberg et al. 2005).

The lack of HLA matched donor due to small families is the limitation in HSCT. However, preimplantation genetic diagnosis (PGD) facilities by umblical cord blood as an alternative source of hematopoietic stem cells provide a novel option in such case (Bielorai et al. 2004, Grewal et al. 2004; Verlinsky et al. 2001). The use of PGD for combined analysis of mutation and HLA matching enables the birth of an unaffected child who can serve as a potential donor for an affected sibling in need for stem cell transplantation. PGD uses standard assisted reproduction technologies and requires the biopsy of material from either the oocyte and/or the developing embryo. The biopsied material tested for the genetic defect and HLA match unaffected embryos is transferred to the uterus. At the time of birth of a child, cord blood cells are harvested and used for HSCT. However, the technique has some disadvantages such as low success rate, health risk and financial and emotional burden involved with the procedure. In addition, a number of families undergoing PGD had experienced repeated unsuccessful PGD cycles (Schindler and Hoehen 2007).

Thus, despite recent advances in the BMT that have lead to an increase in the overall survival rate of FA patients, there is still a need to establish new therapies for proper and cost effective treatment of FA.

## Future therapies

### Gene Therapy

Transduction of fibroblasts, lymphoblastoid cells, or hematopoietic cells from FA patients using retroviral vector containing the cDNA for *FANCA* has shown that ex-vivo gene transfer is feasible as a treatment for the hematopoietic defects observed in FA (Cohen-Haguenauer et al. 2006). However, to increase the efficiency of gene transfer, it requires sufficient numbers of hematopoietic stem/progenitors cells from FA patients that are difficult to obtain (Croop et al. 2001). Moreover, ex vivo culture for gene transfer is deleterious to FA cells and may be responsible for the poor success rate of earlier gene therapy trials in FA patients (Aube et al. 2002; Habi et al. 2005; Liu et al. 1999). FA patient cells do not survive well in ex vivo culture due to their sensitivity to oxygen mediated damage, though the cells successfully grow in a low oxygen environment. Clinical trial of retrovirus-mediated gene transfer in *FANCA* patients was not successful, because durable engraftment of gene-modified cells could not be achieved (Kelly et al. 2007). Recent studies demonstrating gene therapy using lentiviral vectors seems to be promising due to shorter transduction time, minimizing ex vivo culture, and high level transgene expression in hematopoietic cells. Another advantage of lentiviral vectors is that they do not integrate close to the promoters of transcriptionally active genes as frequently as gammaretroviral vectors do, and therefore reduces the insertional mutagenesis, and the risk of vector-mediated dysregulation of nearby genes (Beard et al. 2007). Various studies of gene therapy using lentiviral vectors have been reported. Becker and co-workers demonstrated that lentiviral transduction of CD34 cells, with ex vivo cell culture in the presence of low oxygen (5%), reducing agent N-acetyl-L-cysteine (NAC), and a combination of growth factors improves viability and engraftment potential of human cells (Becker et al. 2010). Habi and group have demonstrated that long-term expression of the *Fancc* transgene can be achieved by a direct in vivo gene transfer approach using lentiviral vector to deliver the *Fancc*-EGFP transgene. Transduced stem and progenitor cells were functionally corrected for their MMC sensitivity thus showing that the inserted transgene is functional in correcting this defect. In addition, mice intrafemorally injected with the *Fancc-EGFP* transgene were resistant to the MMC-induced progressive BM failure (Habi et al. 2010).

Future trials of gene therapy are strengthened by the fact that some FA patients are mosaic where a subgroup of cells is negative for DEB/MMC test while others are positive. Somatic mosaicism occurs due to the reversion of a pathogenic allele to 'wild' type in a single somatic cell and leads to the generation of normal functional allele, resulting in the growth/survival advantage of the corrected cell in the background of FA cells. The clinical implications of revertant mosaicism depend on the developmental stage, blood cell lineage affected by the reversion event and the clinical situation of the mosaic patient (Hoehn et al. 2007). In self renewing tissues, the reversion event may convey a growth advantage to the revertant cells leading to the ultimate replacement of the defective cells. The mechanisms of the natural gene therapy vary and involve mitotic crossover, gene conversion, back mutation or second site mutation (Hoehn et al. 2007). Depending on the mechanism of somatic reversion, the function of affected cell lineage may be partly or completely restored (Gross et al. 2002).

In a recent review, International FA Gene Therapy Working Group has proposed an optimal strategy for conducting stem cell gene therapy clinical trials in FA (Tolar et al. 2011). The group has recommend HIV-1 derived self inactivating lentiviral vectors as the most suitable vector for initial phase I clinical trial for gene correction in fanconi anemia. For FANCA gene therapy, Phosphoglycerate kinase (PGK) promoter in combination with an optimized woodchuck hepatitis virus posttranscriptional regulatory element (WPRE) based lentiviral vectors are useful since the combination ensures stable transgene expression and low genotoxicity. The group advises use of purified grafts from FA bone marrow, or mobilized peripheral blood cells and recommends for hematopoetic harvest at early stages of the disease to obtain sufficient numbers of hematopoietic stem/progenitors cells. Major inclusion and exclusion criteria for gene therapy clinical trial in patients with bialleleic FANCA germ line mutations were also proposed (Table 1). Conducting multi-centre gene therapy protocols using this design will further provide a framework for establishing next generation clinical trials using various modifications.

**Table 1:** Major inclusion and exclusion criteria for gene therapy in patients with biallelic FANCA germ-line mutations as proposed by International Fanconi Anemia Gene Therapy Working Group.

Inclusion Criteria	1. FA demonstrated by a positive test for increased sensitivity to chromosomal breakage with MMC/DEB and determination of FA complementation group A by somatic cell hybrids, molecular characterization, western blot analysis, direct FANCA sequencing, or acquisition of mitomycin C resistance after in vitro transduction with a vector bearing the FANCA cDNA.
	2. Bone Marrow analysis demonstrating normal karyotype.
**Exclusion Criteria**	1. Uncontrolled infection (viral, bacterial, or fungal).
	2. Patients with an HLA identical sibling donor.

Recently, generation of patient-specific induced pluripotent stem (iPS) has shown a great therapeutic potential to obtain disease corrected patient specific cells (Raya et al. 2009). This observation has wider applications in cell and gene therapy and can help in overcoming the limitation of gene therapy, insertional oncogenesis and lack of hematopoietic stem cells. Like embryonic stem (ES) cells, human iPS cells can differentiate to any cell lineage in the body; and genetically corrected iPS cells specifically target safe integration sites of the therapeutic transgenes. Raya et al in 2009 showed that on the introduction of wild-type transgene, reprogramming efficiency is restored but endogenous FA mutation carrying iPS cells may be generated and subsequent knockdown of the correcting transgene led to rapid loss of self-renewal. This suggests that an intact FA pathway is required for induction and maintenance of pluripotency (Raya et al. 2009). However, it may be possible that disruption of the DNA repair machinery in FA cells precludes resolution of the DNA breaks that occurs due to the reprogramming protocol with retroviral/lentiviral integration. Thus using nonintegrating transgenes for reprogramming may permit the generation of genetic lesion free FA iPS cells and can also be propagated continuously. Further studies will be required to elucidate the precise role of the FA pathway in induction and maintenance of pluripotency (MacMillan et al. 2011). Other limitations of IPS cells that need to be addressed are chromosome abnormalities, differences in gene expression and epigenetic state, obtaining pure cell population and immunologic rejection in autologous transplantation in mice (Nordin et al. 2011).

### New emerging Therapies

Small molecule intervention, targeting TLR8 pathway to suppress TNFα production and profiling DNA damage repair pathways are new exciting emerging therapies under research.

Traditional methods have been successful in preventing anemia however they could not prevent the occurrence of cancer at later stages of life in FA patient. Thus a small molecule that can prevent both anemia and cancer will be an ideal therapy for FA. The best solution will be the prevention of DNA damage or DNA repair. Since FA cells are known to be hypersensitive to reactive oxygen species (ROS) that can damage the DNA and therefore cause mutation and cancer (Du et al. 2008; Muller and Williams 2009), treatment with antioxidants can reduce oxidative DNA damage and thus has the potential to delay cancer in FA. First small molecule tested in FANCD2 mice was Tempol (4-hydroxy-2,2,6,6 tetramethyl piperidine-N-oxyl), a nitroxide antioxidant and a superoxide dismutase mimetic; it was found to delay epithelia tumor onset (Zhang et al. 2008). Moreover, it did not adversely affect the repopulating capability of FA hematopoietic stem cells. Treatment with the antioxidant drug, resveratrol, an activator of SirT1 deacetylase, was demonstrated to partially correct hematopoietic defects in Fancd2^-/-^ mice (Zhang et al. 2010). Similarly, other molecules such as N-acetyl cystine and chloroquine may also be tested for their therapeutic potential in treating FA.

By High-Throughput cell screening, potential small molecule inhibitors of FA/BRCA pathway such as wortmannin, H-9, and alsterpaulone and curcumin were identified (Chirnomas et al. 2006) in which curcumin, a natural compound was found to be most suitable inhibitor because it is less toxic and thus safe to administer.

Another developing field of investigation for FA therapy is TLR8 pathway dependent TNF-α overproduction. TNFα, a molecule which plays a direct role in the pathogenesis of both bone marrow failure and clonal evolution in Fancc^-/-^ mice (Li J. et al. 2007). In marrow failure, hypersensitivity to TNFα is exacerbated by the overproduction of TNFα by FA cells, (Dufour C et al. 2003) which is controlled by both transcriptional and posttranslational mechanisms that require activation of MAPK pathways (Briot D et al. 2008). It was found that TLR8 (or a TLR8-associated protein) is highly ubiquitinylated in mutant FA-C cells and that overproduction of TNFα in mutant cells depends on TLR8. Studies using THP-1 blue shFANCC cells indicate that a major mechanism by which p38 influences TNFα production is by suppressing TNFα gene transcription. Other potential binding partners of TLR8 include MyD88, Mal, IRAK4, IRAK1, and other TLRs (TLR7 and TLR9) (O'Neill LA. 2008). TLR8 may also bind to negative regulators, SIGIRR, ST2L, MyD88s, SOCS1, Tollip, IRAKM, IRAK2c/d, and TRIAD3A. All these proteins should be considered as possible targets for this key posttranslational modification. Thus TLR8 pathway represents a potentially attractive developmental therapeutic target in FA for high-throughput screening for molecules that activates/inactivates this pathway in FA-C cells, and the THP-1 Blue/FANCC shRNA cells provide a convenient tool for the screening.

FA patients have an increased risk of developing tumours, myelodysplastic syndromes (MDS) and acute myeloid leukaemia (AML), suggesting that the FA proteins take part in DNA damage repair. Profiling of DNA repair pathway through synthetic lethality mechanism can be applicable as therapeutic approach for FA pathway deficient tumours. There are various DNA repair pathways; base excision repair (BER), nucleotide excision repair (NER), mismatch repair (MMR), homologous recombination (HR), nonhomologous end-joining (NHEJ), and translesion DNA synthesis (TLS) in the cell that plays a role when DNA damage occurs, depending upon its type. All FA proteins interact in a common pathway involving in homologous recombination. Loss of one DNA repair pathway may result in hyper-dependence on a second, compensatory DNA repair pathway. For example, cancer cells with a mutated FA pathway may be hyperdependent on the BER, NHEJ or TLS pathway (D'Andrea 2010; Tutt et al. 2001). Therefore, in FA patients, tumours with defect in FA pathway can be treated by inhibiting this second pathway. The idea is to find a drug that can target the second pathway and can effectively kill the cancer cell. For example, FA deficient tumour cells are known to be hypersensitive to the inhibition of CHK1, particularly when combined with cisplatin therapy (Chen et al. 2009). Moreover, if the FA pathway serves as a backup for some other pathway, inhibition of such a pathway will lead to the activation of the FA pathway and thus be detrimental in FA pathway deficient cells (Evers et al. 2010). For example, Poly-ADP-ribose polymerase-1 (PARP-1) is involved in base excision repair, a pathway repairing DNA single-strand breaks (SSB). Loss of PARP1 leads to the conversion of SSBs to double-strand breaks (DSBs) and increases the demand for HR repair pathway. Utilizing PARP inhibitors may therefore be a successful therapeutic strategy for a subset of AML patients with deficient in HR such as FA deficient pathway or deficient in RAD51 loci (D'Andrea 2010).

In future, identification of the new partners of the FA pathway as well as other DNA damage repair pathways, understanding the crosstalk between these pathways and the mechanism of their regulation will help in generating new small inhibitor molecules that can act as drug target in devising a promising therapeutic strategy for targeting DNA damage repair pathways.

To conclude, since the FA clinical trials are undergoing and research for a better therapeutic approach is underway, so in the present scenario, a proper algorithm based on the available established treatments and recent scientific advances should be devised for the treatment of the FA. If the clinical diagnosis has been done on the basis of physical abnormalities, bone marrow failure, haematopoiesis and organ abnormalities or cancer, first step should be to perform the chromosome breakage test. If the test is positive, molecular analysis through FANCD2 western blot can be done to confirm the diagnosis and to classify the patients according to FA/BRCA pathway. The patient should then be tested for complementation analysis and mutation testing. Relatives also need to be checked for chromosome fragility and mutation analysis. Histocompatibility antigen (HLA) typing should be done for stem cell transplantation. If HLA matched and non FA donor is available in the family, HSCT can be the first line of action. However, as the risk of graft failure increases in transfusions, transplantation should be performed before the patient is transfused. If HLA matched donor cannot be found then search for unrelated HLA matched donor should be started. Until the donor is found, androgens and growth factors will be good holding treatment to delay haematopoietic stem cell depletion. If the search for unrelated donor is still not successful, then in vitro fertilization, PGD and HSCT using umbilical cord blood collection may be a choice. However, it should be noted that HSCT treatment using HLA matched or unrelated donor has shown only marginal difference in the survival rate of the patient. Three year overall survival after HSCT in recipients of FA-negative HLA matched sibling grafts was 69-93% whereas using alternative donor grafts survival was 52-88%, (MacMillan et al. 2010; Wagner et al. 2007;Farzin, et al. 2007). Finally, gene therapy can also be considered for the treatment.

## Authors’ original submitted files for images

Below are the links to the authors’ original submitted files for images. Authors’ original file for figure 1
