# Supplementary figures and images for: Current and emerging therapeutic strategies for Fanconi anemia
Source: Hugo J. 2012 Mar 9;6(1):1. doi: 10.1186/1877-6566-6-1 (PMC4685155; doi:10.1186/1877-6566-6-1)

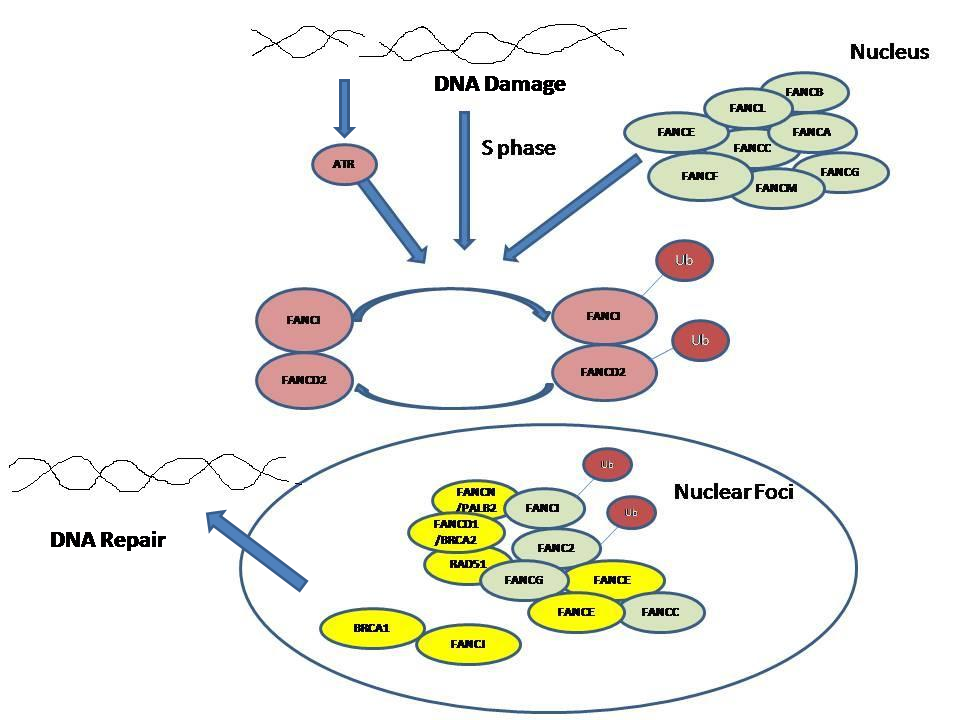

Supplement: Supplementary file 1 — Authors’ original file for figure 1 [file 11568_2011_1_MOESM1_ESM.tiff]
